# Supplementary material for: Vaccinating adolescents against SARS-CoV-2 in England: a risk–benefit analysis
Source: J R Soc Med. 2021 Nov 1;114(11):513–24. doi: 10.1177/01410768211052589 (PMC8649477; doi:10.1177/01410768211052589)
Supplement: sj-pdf-1-jrs-10.1177_01410768211052589 - Supplemental material for Vaccinating adolescents against SARS-CoV-2 in England: a risk–benefit analysis [file sj-pdf-1-jrs-10.1177_01410768211052589.pdf]

Vaccinating adolescents against SARS-CoV-2 in England: a risk-benefit analysis

Deepti Gurdasani[1] PhD†, Samir Bhatt PhD[2], Anthony Costello FMedSci[3], Spiros Denaxas PhD[3], Seth Flaxman PhD[2], Trisha Greenhalgh PhD[4], Stephen Griffin PhD[5], Zoë Hyde PhD[6], Aris Katzourakis PhD[4], Martin McKee DSc[7], Susan Michie PhD[3], Oliver Ratmann PhD[2], Stephen Reicher PhD[8], Gabriel Scally FFPHM[9], Christopher Tomlinson MBBS[3], Christian Yates PhD[10], Hisham Ziauddeen PhD,[11] Christina Pagel PhD[3]†

† Corresponding authors:
Prepublication
Martin McKee martin.mckee@lshtm.ac.uk
Post publication
Deepti Gurdasani d.gurdasani@qmul.ac.uk
Christina Pagel c.pagel@ucl.ac.uk

Abstract

Objective

To offer a quantitative risk-benefit analysis of 2 doses of SARS-CoV-2 vaccination among adolescents in England.

Design

Following the risk-benefit analysis methodology carried out by the US Centres for Disease Control (CDC), we calculated historical rates of hospital admission, ICU admission and death for ascertained SARS-CoV-2 cases in children aged 12-17 in England. We then used these rates alongside a range of estimates for incidence of Long COVID, vaccine efficacy and vaccine-induce myocarditis, to estimate hospital and ICU admissions, deaths and cases of Long Covid over a period of 16 weeks under assumptions of high and low case incidence.

Participants

All 12-17 year olds with a record of confirmed SARS-CoV-2 infection in England between 1st July 2020 and 31st March 2021 using national linked electronic health records, accessed through the British Heart Foundation Data Science Centre.

Main outcome measures

Hospitalisations, ICU admissions, deaths and cases of Long COVID averted by vaccinating all 12-17 year olds in England over a 16 week period under different estimates of future case incidence.

Results

[1] Queen Mary University of London, UK
[2] Imperial College London, UK
[3] University College London, UK
[4] University of Oxford, UK
[5] University of Leeds, UK
[6] University of Western Australia, Australia
[7] London School of Hygiene and Tropical Medicine, UK
[8] University of St. Andrews, UK
[9] University of Bristol, UK
[10] University of Bath, UK
[11] University of Cambridge, UK

At high future case incidence of 1000/100,000 population/week over 16 weeks, vaccination could avert 4,430 hospital admissions and 36 deaths over 16 weeks. At the low incidence of 50/100,000/week, vaccination could avert 70 hospital admissions and 2 deaths over 16 weeks. The benefit of vaccination in terms of hospitalisations in adolescents outweighs risks unless case rates are sustainably very low (below 30/100,000 teenagers/week). Benefit of vaccination exists at any case rate for the outcomes of death and long COVID, since neither have been associated with vaccination to date.

Conclusions
Given the current (as at 15 September 2021) high case rates (680/100,000 population/week in 10-19 year olds) in England, our findings support vaccination of adolescents against SARS-CoV2.

Introduction

On 19th July 2021 the Joint Committee on Vaccination and Immunisation (JCVI), which advises on vaccine policy in England and Wales, recommended that COVID-19 vaccines should not be offered to all 12-17 year-olds, judging that any health benefits relative to potential risks were marginal.[1] This decision was made even though the UK's Medicines and Healthcare Products Regulatory Agency (MHRA), having sought expert advice from the independent Commission on Human Medicines, had approved the use of the Pfizer/BioNTech vaccine for those aged 16 years and above in December 2020, and for 12-15 year-olds on 4th of June 2021.[2] JCVI initially recommended that vaccines should only be offered to those under 18 who were either living with immunosuppressed household members or had one of a specified set of pre-existing conditions, later updating this position[3] by offer a first dose of vaccine to all 16-17 year olds, with a decision on second doses delayed pending further assessment by the JCVI. Subsequently, on 3rd September,[4] the JCVI further updated their advice suggesting that risks and benefits of vaccination were finely balanced,[4] stating that the majority of the more severe outcomes occurred in children with pre-existing conditions, among whom the risk, and therefore the potential benefit, appeared to be higher.[4] However, their analysis did not assess the risk posed by long COVID, which can occur in all children, and can lead to persistence of symptoms in 2-14 % of children for 4-16 weeks or longer.[5-10] Nor did it assess benefits to children through reduction of educational disruption, reduced risk to household members, or broader societal benefits through reduction in overall transmission due to higher population immunity.[4] Based on their assessment, JCVI did not recommend routine vaccination of adolescents.[4] On the 13th September[11] based on the assessment by the JCVI, and additional consideration of direct benefits to children from reduced educational disruption, the four UK Chief Medical Officers recommended a single dose of vaccine for all 12-15 year olds, pending further assessment.

The JCVI position and UK policy contrasts with the policies of many other countries, including the US, Canada, Australia, New Zealand, Israel, and much of Europe and Southeast Asia, that are currently offering two doses of vaccines to all 12–17 year-olds. The European Medicines Agency (EMA) has authorised both the Pfizer/BioNTech (Comirnaty) and Moderna (Spikevax) vaccine for 12–17 year-olds.[12-13] To date, the US has fully vaccinated 10 million under-18s, including over 6 million 12–15 year-olds, and has administered at least one dose to over 12 million under-18s, including almost 8 million 12-15 year olds.[14] France has already fully vaccinated 52 % of its 12-17 year olds and the majority of adolescents have received at least a single dose in Denmark and Spain.[15]

The USA was one of the first countries to offer vaccines to adolescents.[16] This decision was based on quantitative analysis of the potential risks and benefits of vaccination in children conducted by the US Centres for Disease Control and Prevention (CDC)[6], considering in particular the risk of myocarditis and pericarditis.[17-20] These have a rare association (30-40 cases/million doses) with COVID-19 vaccination,[17,20] particularly following second doses. Thus far, as per the joint statement issued by the CDC, the American Academy of Paediatrics, and several other organisations, almost all cases of vaccine attributable myocarditis/pericarditis in young people have been mild, and have recovered with no or minimal treatment.[18-19,21-22] There have been no vaccine-related deaths recorded and no serious adverse events observed in over 10 million under 18s vaccinated to date. The UK analysis of Yellow Cards (notifications of adverse events) by the MHRA, also states that these events have been extremely rare, typically mild, and with rapid recovery.[23] On the other hand, COVID-19 illness can be associated with myocarditis[18,24-27] and hospitalisation has been associated with long-term neurological impacts, even in those under 18 years of age.[28] Based on their quantitative analysis, the CDC concluded that the potential benefits did outweigh the risks[16-17], and recommended vaccination for all children aged 12-15.[16] In their analysis, the CDC used rates of infection in the US from 21st May 2021, assuming these would remain constant over the following 120 days, and used a hospitalisation rate of 0.8/100,000 population/week.[17]

Analysis by the UK Joint Committee on Vaccination and Immunisation (JCVI)
The publicly available versions of the JCVI recommendations contain limited quantitative analyses of the benefits and risks to 12-17 year olds.[1,3,4] In their first statement on the 19th July, they estimated a COVID-19 attributable mortality rate of 2 per million in England and hospitalisation rate of 100-400 per million (during the second wave) in the UK among children and young people (under 18 years of age). They did not give a denominator but these estimates appeared to use the whole population of under 18s in England (30 deaths in just over 12 million children), rather than using the number of reported COVID-19 cases or an estimate of overall infections as the denominator. The use of rates based on the total population substantially underestimates the risks of these outcomes in children infected with SARS-CoV-2, unless it is assumed that every child has been infected. This is both implausible and inconsistent with data on antibody prevalence.[1]

The most recent JCVI statement published on 3rd September 2021, while providing numbers for risk of ICU admission to healthy 12-15 year olds, and estimates of hospitalisations and ICU admissions averted, does not clarify what hospitalisation rates were assumed, what the denominator was for ICU admission rate, how this modelling was carried out or what future infection exposure was considered over what period of time.[4] Neither does the analysis consider the impact of long COVID, a clinically significant complication of SARS-CoV-2 infection that occurs in somewhere between 2% and 14% of cases, even in healthy children.[5-10] The longer term consequences of acute COVID-19 infection are also not yet known, but emerging studies show persistent changes in multiple organs,[27] including the brain,[28-29] heart,[24-26] lungs and kidneys.[27]

COVID-19 outcomes depend on risks of exposure, rather than total population size. Exposure to infection varies considerably over time and depends on context such as vaccination coverage and protective public health measures. The higher exposure is, the greater the benefit of vaccination (as it averts more cases for the same number of people vaccinated). Thus any risk-benefit analysis must consider potential benefits of vaccination at different levels of exposure to infection, as done by the CDC.

Current context in England
June to August 2021 saw very high case incidence rates in 12 to 17 year olds (500 to 1,000 cases per 100,000 people per week).[30] Official mandates for nearly all mitigations, including mask mandates, social distancing and self-isolation requirements for vaccinated adult contacts and children under 18 have been removed in England. There have been over 3,400 hospitalisations in under 18s with COVID-19 since 4th June (when the Pfizer vaccine was approved for 12-15 year olds by MHRA), including over 1,700 hospitalisations among 6-17 year olds[31], with the majority of these directly attributable to COVID-19 (PHE CHIME and ISARIC).[32-33] Case rates fell in under 18s after the end of the summer school of term but have been increasing again since August.[34] Hospital admissions in 6-17 year olds remain at levels near the winter peak (**Figure 1**). Government advisors have warned that significant increases[35] in infections are likely following the recent return to school in September.

Objective
In this paper, we offer a quantitative assessment of the benefits and risks of vaccination in 12-17-year-olds for England over a range of case incidence rates. To be consistent with JCVI remit, we consider only direct benefit to children from vaccination, and do not consider secondary benefits such as onward transmission or educational disruption.

Methods
Using the CDC analysis as a template,[17] we examined the potential benefits and risks of offering vaccines to England's 3.9 million 12-17-year-olds ahead of school reopening in September 2021. To do this we extracted data on the number of 12-17-year-olds in England diagnosed with COVID-19 and

the related hospitalisations and deaths, during the period from 1st July 2020 to 31st March 2021. This period was chosen to exclude the first wave of the pandemic when few children were tested for SARS-CoV-2. Linked data since 31st March 2021 are not yet available. Data were obtained using linked electronic health records from multiple sources (accessed through the British Heart Foundation Data Science Centre).[36] We used information from: a) the Public Health England (PHE) Second Generation Surveillance System (SGSS) national testing laboratory, b) primary care consultations in General Practice Extraction Service Data for Pandemic Planning and Research (GDPPR), c) hospitalisations (including ICU admissions and ventilation care provided outside of an ICU) from Hospital Episode Statistics (HES) and the COVID-19 Hospitalisations in England Surveillance System (CHESS), and d) deaths from Office of National Statistics (ONS). Patients were included in the analyses if they resided in England, were alive on the start date of the study period, registered with a primary care practice, had a valid pseudo-identifier for linkage and at least 28 days of follow up. Using this individual-level linked dataset, we calculated the proportion of hospitalisations, ICU admissions and deaths associated with identified infection (**Table 1**).

Using these proportions and varying future COVID-19 case incidence rates, we estimated the number of COVID-19 related hospitalisations, ICU admissions and deaths expected over the 16 weeks from September to December 2021, and the number of cases of long COVID that would arise, without vaccination. We defined Long COVID broadly as persistent symptoms following acute infection for 12 weeks or more. In line with this definition, we considered three estimates based on recent studies on Long COVID: 2% (Molteni et al),[10] 4% (Radke et al)[7] and 14% from the large-scale CLoCk study.[6] We note that these estimates encompass the estimates recently reported by the Office for National Statistics (ONS), which identified self-reported long COVID prevalence of 6%[5] among 12-16 year olds. Differences in estimates likely arise from different symptom lists, time gaps allowed between symptoms, treatment of missing data, case ascertainment, and time spans for determining Long COVID. Using a broad range of estimates allows for the uncertainty in the prevalence of long COVID, and allows us to ascertain benefit across a range of possible scenarios.

We use ascertained case rates (which do not capture all infections since they rely on a positive test) to calculate historical rates of adverse outcomes and to project forward. We chose this approach as PHE incidence data reported in England is based on ascertained cases,[30] and data on ascertained cases is more robust and can be linked at an individual level to outcomes. Using the same denominator consistently as we have done would produce the same estimates of hospitalisations and deaths as using infection rates, infection fatality and infection hospitalisation rates. However, this does assume that the proportion of infections ascertained as cases through testing remains relatively constant over time. To assess the impact of violations of this assumption, we also carried out a sensitivity analysis using lower hospital admission to case ratios to generate outcomes allowing for the possibility that case ascertainment has improved over time.

Given the projected estimates are based on recent and current per-population case incidence rates per week, as reported weekly by PHE, these implicitly account for the proportion of children susceptible to SARS-CoV-2 which is not expected to change markedly over a period of 16 weeks, even in our high incidence scenario, thereby eliminating the need to explicitly model susceptibility during the 16 week period.

We used CDC estimates of vaccine associated myocarditis/pericarditis following the first and second doses of vaccine,[17-20] assuming all 12-17 year-olds in England were vaccinated. These are the same estimates that JCVI refer to in their statement, and are compatible with yellow card reporting from the MHRA. Next, we examined how many COVID-19 related outcomes would be averted by vaccination of all 12–17-year-olds, assuming conservative estimates of vaccine effectiveness in reducing severe outcomes (90% with the Delta variant among the fully vaccinated) and infections (64%

among the fully vaccinated),[37-39] assuming no additional protection against long COVID once infected. We calculated total hospitalisations averted assuming the worst-case scenario of all cases of vaccine associated myocarditis requiring hospital admission.

Given the difficulty in predicting infections over time, we examined outcomes across a broad range of average future ascertained case incidence rates over a fixed period of 16 weeks. Full outcomes are shown for two levels of exposure: late July 2021 ascertained case rates in 12-17 year olds of 1,000 per 100,000 population per week[30] for 16 weeks and a 20-fold lower level of exposure of 50 per 100,000 population per week for 16 weeks, comparable to the end of April 2021. We note that the high incidence scenario is possible given case rates in Scotland reached 1,800 per 100,000 population per week in all children under 15[40] and 2,700 per 100,000 population per week[33] in 14 to 15 year olds in early September 2021 following school opening in mid-August. Approximately 5 % of all under 15s in Scotland have had a new confirmed infection in the month since school opening[40].

We also calculated expected hospital admissions across the full range of ascertained case rates from 0 to 1,000/100,000/week, to find the minimum ascertained case incidence threshold at which benefit tilts from no vaccination towards vaccination.

In presenting results, we round all estimates higher than 50 to the nearest 10 or to two significant figures if more than 1,000.

Results

We estimated that, if the late July 2021 rates of infection among 12-17 year olds (1,000 per 100,000 per week) continued over 16 weeks in England, this would lead to 5,100 hospitalisations, 340 admissions to ICU (with 280 adolescents requiring ventilation), and 40 deaths. Vaccination is estimated to avert 4,590 COVID-19 hospitalisations, 310 ICU admissions, 250 needing ventilation, and 36 deaths, with the disbenefit of 160 cases of vaccine-associated myocarditis/pericarditis (see **Figure 2A**). Under the assumption that all 160 cases of vaccine-associated myocarditis/pericarditis required hospitalisation, vaccination would still avert 4,430 hospitalisations. For long COVID, vaccination would avert 56,000, 16,000, or 8,000 cases in 12-17 year olds assuming incidence rates of 14%, 4%, and 2% respectively.

Examining the low-incidence scenario of 50 per 100,000 per week, vaccination could avert 230 hospitalisations, 15 admissions to ICU, 2 deaths, and 2,800/800/400 cases of long COVID (14%/4%/2% incidence respectively), at the same cost of 160 cases of vaccine-associated myocarditis/pericarditis (See **Figure 2B)**, averting a total of 70 hospitalisations. The risk of hospitalisation with vaccination only exceeds the risk of hospitalisation with COVID-19 when the case incidence is below 30 per 100,000 per week; a level that has not been seen in adolescents in the UK in 2021 (**Figure 3A**). Due to the differential risk of vaccine-related myocarditis in boys and girls, this threshold is 50 per 100,000 per week for boys and below 10 per 100,000 per week for girls (**Figure 3A**). Even at these low incidence rates, vaccines would still provide protection against death (**Figure 3B)** and long COVID outcomes (**Figure 2B**).

We note that our analysis remains robust in the face of substantial changes in the parameters examined. For example, even if we assume that case ascertainment has improved among 12-17 year olds in more recent periods due to increased testing (e.g. with rapid tests) resulting in lower ascertained case hospitalisation rates, a sensitivity analysis assuming a 0.50 % hospitalisation rate (instead of 0.82 %) suggests the overall benefit-risk when comparing only hospitalisations would still be in favour of vaccination down to an incidence of 60/100,000/week.

Limitations
We have explicitly not factored vaccine uptake into our analysis because we only considered direct risks and benefits to children either with or without vaccination. Thus our risk/benefit calculation amongst those vaccinated is not changed by considering vaccine uptake (as both risks and benefits would change by an equivalent amount). Were we considering additional secondary impacts on transmission, vaccine uptake would be a crucial parameter. We note that considering secondary impacts of vaccination (e.g. on transmission or educational disruption) would further tip the balance towards vaccination.

There will also be some children identified as a COVID-19 hospital admission where their positive test is an incidental finding. Addressing these limitations involves challenging analysis which we hope to undertake in the near future, but the decisions on vaccinating 12-17 year olds are pertinent now and we note that neither the CDC, nor similar European agencies have factored such considerations into their analysis and advice. We also note that our analyses would be robust to substantial changes in hospitalisation rates, as we have shown in our sensitivity analyses. Finally, recent reports from SAGE suggest that 80% of hospital admissions in children with COVID-19 were due to COVID-19.[33]

It is likely that there is some difference in risk of hospitalisation in children with and without other health conditions. Unlike JCVI, we have not considered sub-analysis by whether children had pre-existing conditions, due to lack of available data. However, whilst risk of hospitalisation may be much lower in children without other health conditions, we note that the majority of hospital admissions with COVID-19 and PICU admissions with PIMS-TS in children have occurred in children without pre-existing conditions.[41-43] Moreover, the risk of long COVID is likely to be similar in healthy children (given the estimates from the ONS),[5] and our current analysis of benefits with respect to reduction in long COVID risk would apply to this group. Therefore, alongside analyses by JCVI that show benefit in reduction of ICU admissions, hospitalisation and PIMS-TS[4] our analyses would strongly favour benefits of vaccination when long COVID outcomes are also considered in this group.

We do not assess PIMS-TS separately in our study. We expect that, given the majority of cases are admitted to PICU, these would have been captured in our estimates for ICU admission. However, we may have underestimated cases of PIMS-TS in our modelling. This would lead to more conservative results, thereby underestimating the benefits of vaccines relative to risks.

Given the uncertainty in long COVID incidence among adolescents, we have based our assessment of risk and benefit on a range of estimates based on recent studies of long COVID.[5-10] We note that even with the most conservative estimates (2 % at 12 weeks), there are considerable benefits in prevention of long COVID from vaccination, particularly given current incidence rates in England. Notably, the benefits of vaccination remain clear for ascertained case incidence rates of over 30/100,000/week even without factoring in long COVID.

We do not assess risks separately for one dose and two doses of vaccines, given the considerable uncertainty around the durability, and level of protection offered by a single dose of vaccine among adolescents. We note that there have been no clinical trials in adults or adolescents with a single dose of Pfizer. Our analysis shows that benefits of two doses of vaccines, which are likely to be needed to provide maximally effective and durable protection from infection with the Delta variant of SARS-CoV-2, far outweigh risk from vaccine associated myocarditis.

In reality, case rates will not be constant over 16 weeks and so our calculations should not be taken as predictions in any sense. However, our analysis shows that unless there is good reason to believe that average ascertained case rates will be below 30/100,000/week for the entire period, then vaccination will bring a benefit. Thus our analysis provides a realistic benchmark to assess vaccine

policy. We also note that the time frame of 16 weeks is somewhat artificial and children will likely continue to be exposed to COVID-19 well into 2022, which would lead to further benefit from vaccination.

Finally, we have not considered the broader logistical factors involved in vaccination of adolescents in our risk-benefit analysis. This would require further consideration, with analysis of capacity and resource requirements to expand the current programme to these groups.

Discussion

Direct benefit to children

Our analysis shows that vaccination of 12-17-year-olds provides overall benefit in terms of hospital admissions for average ascertained case rates of over 30 per 100,000 children aged 12 to 17 per week. When considering additional outcomes such as deaths and long COVID, vaccination is always beneficial as neither adverse outcome has been linked to vaccination. Since ascertained case rates in 12-17 year olds have not been as low as 30/100,000/week in 2021[30] in England and given likely rates of at least 20 times higher this next school term (current rates in England are 680/100,000/week in teenagers)[30], we conclude that on clinical risks alone, vaccination is warranted for 12-17 year olds in England. SARS-CoV-2 is a neurotropic and pro-inflammatory virus with neuro-invasive potential; structural changes in brain tissue have been observed in adults, including those with mild infection.[29] Long COVID can be associated with multisystem disease in some children, including myocarditis,[24-26] and persistent cognitive symptoms. Myocarditis has been shown to be 37 times more common in under 16s with COVID-19 compared to those without infection.[24] While we wait to understand the long-term effects of SARS-CoV-2 upon children, the precautionary principle advocates for protecting all children from exposure to this virus, and vaccination is a crucial part of that protection where use is authorised. Additionally, previous data from England also shows that while children with pre-existing illnesses may be at greater individual risk, 60 % of hospitalisations in under 18s in England have been amongst children who do not have such conditions,[44] suggesting considerable benefits for all children in reducing severe illness through vaccination.

We note that the assumptions and estimates used in our analysis are conservative. The rates of the outcomes of interest are based on data prior to 31st March 2021 - before the potentially more severe[45] Delta variant became dominant. Estimates from Public Health England and Public Health Scotland suggest that the rate of hospitalisation with Delta is likely to be 1.5-2x greater than that with the Alpha variant, although whether this increased severity is seen for children is not clear.[46] Current data from the USA suggest that indicators of severe disease among hospitalised children during an early period when the Delta variant predominated were generally similar to those observed earlier in the pandemic.[47] A much higher number of hospitalisations may been observed potentially due to greatly increased case numbers as a result of the markedly increased transmissibility of the Delta variant. We have used estimates of vaccine effectiveness that are more conservative than those published by PHE.[48] Consistent with our analyses, hospitalisations among unvaccinated adolescents have been 10 times higher than those among vaccinated adolescents in the USA.[47] In line with the CDC approach, we have considered the risks and benefits over a limited time period. Using less conservative estimates of efficacy or projecting over longer time periods will further favour vaccination.

Though our analysis is based on data from England, the same calculations can be made for other countries, using appropriate country-specific data. To assist this process, we have made our calculations spreadsheet publicly available (see Spreadsheet) for scrutiny and for widespread use. We welcome such scrutiny and invite the JCVI to examine our analysis. We also invite JCVI to similarly make available the data and calculations on which they have based their own conclusions.

Wider benefits of vaccination

As discussed, we have only considered the direct impacts of vaccination on health outcomes in 12-17 year olds, to be consistent with the approach taken by JCVI. The JCVI have not formally evaluated the impact of vaccination upon community transmission.[1] However, given that JCVI has recommended vaccination for 12-17-year-olds living with immunocompromised family members, there is clearly a case for considering the additional benefits in terms of reduced community transmission. Children are a part of the wider community, and vaccinating them is likely to have benefits beyond prevention of childhood illness. Almost 9,000 children in England and Wales have lost at least one primary carer to COVID-19 during the pandemic.[51] The impact of bereavement at an early age cannot be overstated. Vaccinating children will reduce onward transmission to household members, and protect them as well from illness, long COVID, and severe outcomes from COVID-19.

Models examining vaccine roll-out in younger age groups or groups with high levels of contact[49] suggest that vaccination could have substantial impacts upon reducing community transmission, thereby providing much greater protection at population level. Indeed, modelling suggests that vaccinating only a part of the population, while transmission is allowed to continue at high levels, creates conditions permissive for viral adaptation towards potential immune escape.[49,50] Vaccinating adolescents would also protect children and siblings who are not eligible for vaccination, and education staff who interact directly with children in schools. Recent data suggest that 2.3 % and 1.95 % of all education and teaching staff had long COVID symptoms lasting 4 weeks and 12 weeks or more, respectively on the 1st August 2021, the 3rd highest across all occupations examined.[52] Reduction in transmission in schools is likely to have broader protective effects, reducing risk to education staff engaged in face-to-face interactions. While the vast majority of education staff are likely to be double vaccinated, the protection from infection is partial, and reduction in exposure would provide further protection.

Another major consideration is the benefit of vaccination in terms of reducing education disruption for children, given that children testing positive must isolate at home for 10 days and potential impact on education for children who experience symptoms for longer than 10 days. Even mild initial symptoms or Long COVID symptoms that resolve fully after several months could have a significant impact upon children if illness falls during exam periods. Factoring in these various indirect benefits would tilt the balance further in favour of vaccination.

Additional protections alongside vaccination

Immunisations take time and even with this protection, outcomes are better when risk of exposure is lower. England has failed to put in place adequately robust preventative measures in schools thus far[53], and has removed ones that were in place going forward (no mask mandates, minimal contact tracing within schools, no requirement for under 18s to isolate if a household member tests positive). Since community transmission rates remain high and most adolescents were not eligible for vaccination before the start of the new academic year, it is vital that the UK Government invests in mitigations for schools,[53] including assessment and provision of adequate supplemental ventilation. As the CDC has recently emphasised, controlling spread of the Delta variant requires maximum preventive measures including vaccination, masking and ventilation.[54,55] It is not an either-or; a comprehensive 'vaccines plus' approach is needed.

Conclusions

In summary, our conservative analysis shows that the benefits of offering two doses of vaccine to all 12-17 year-olds clearly outweigh the risks to the children concerned in both the current context and in scenarios with substantially lower case incidence rates. The real-world short-term risks from vaccination in over 12 million under 18s who have been vaccinated around the world have been found to be minimal, with myocarditis being a rare, and typically mild complication. The known impact of

COVID-19 on adolescents is concerning, and it is clear that vaccines can avert much of this impact even at current case incidence rates in England.

Competing Interests: CP, MM, SR, GS, AC, SM and KY are members of Independent SAGE. SM is a member of SPI-B. ZH is a member of OzSAGE.

Funding: None

Ethical approval: Data access approval was granted to the CVD-COVID-UK consortium through the NHS Digital online Data Access Request Service (ref. DARS-NIC-381078-Y9C5K). The North East-Newcastle and North Tyneside 2 research ethics committee provided ethical approval for the CVD-COVID-UK research programme (REC No 20/NE/0161). Ethical approval of the CVD-COVID-UK consortium has been described in detail previously.[36]

Guarantor: DG & CP

Contributorship: DG and CP conceived and designed the model, and wrote the first draft of the manuscript. SD extracted and curated the data on adolescent cases, hospitalisations, ICU admissions and deaths. KY, SB and SF contributed to the design of the model, refining, and checking analyses. All authors contributed to the study design, interpretation, manuscript editing, discussion, and contextualisation of this work.

Additional data: A spreadsheet providing the calculations used in this paper is available online. This can be used to calculate parameters for other settings and other times

References

1. Joint Committee on Vaccination and Immunisation. JCVI statement on COVID-19 vaccination of children and young people aged 12 to 17 years: 15 July 2021, 2021.
2. Gov.uk. The MHRA concludes positive safety profile for Pfizer/BioNTech vaccine in 12- to 15-year-olds. 2021.
3. Gov.uk. Independent report: JCVI statement on COVID-19 vaccination of children and young people aged 12 to 17 years: 4 August 2021
4. Gov.uk. Independent report: JCVI statement on COVID-19 vaccination of children aged 12 to 15 years: 3 September 2021
5. Office for National Statistics. Technical article: Updated estimates of the prevalence of post-acute symptoms among people with coronavirus (COVID-19) in the UK: 26 April 2020 to 1 August 2021
6. Stephenson T, Shafran R, De Stavola B, et al. Long COVID and the mental and physical health of children and young people: national matched cohort study protocol (the CLoCk study). *BMJ Open* 2021; 11(8): e052838.
7. Radtke T, Ulyte A, Puhan MA, Kriemler S. Long-term Symptoms After SARS-CoV-2 Infection in Children and Adolescents. *JAMA* 2021.
8. Miller M, Nguyen V, Navaratnam AM, Shrotri M, Kovar J, Hayward AC et al. Prevalence of persistent symptoms in children during the COVID-19 pandemic: evidence from a household cohort study in England and Wales. MedRxiv. 2021.
9. Ministry of Health, Israel. A New Survey. 2021 https://www.gov.il/he/departments/news/childrenpostcoronaeffects
10. Molteni E SC, Canas LS, Bhopal SS, Huges RC, Antonelli M. Illness duration and symptom profile in symptomatic UK school-aged children tested for SARS-CoV-2. *The Lancet Child and Adolescent Health* 2021.

11. Gov.uk. Correspondence: Key published inputs to the UK CMOs advice on universal vaccination of children and young people aged 12 to 15 years against COVID-19. 3rd September 2021
12. European Medicines Agency. COVID-19 vaccine Spikevax approved for children aged 12 to 17 in EU. 2021.
13. Pitchers, C. Pfizer's COVID jab is first in EU to be approved for 12-15 year olds. Euronews. 2021
14. Centers for Disease Control and Prevention. Demographic Characteristics of People Receiving COVID-19 Vaccinations in the United States. 2021.
15. Reed J. Covid: Which countries are vaccinating children and why? BBC News. 2021. https://www.bbc.co.uk/news/health-58516207
16. Centers for Disease Control and Prevention. COVID-19 Vaccines for Children and Teens, 2021
17. Wallace M, Oliver S. COVID-19 mRNA vaccines in adolescents and young adults: Benefit-risk discussion: CDC, 2021.
18. Bozkurt B, Kamat I, Hotez PJ. Myocarditis With COVID-19 mRNA Vaccines. *Circulation* 2021; 144(6): 471-84.
19. Gargano JW, Wallace M, Hadler SC, et al. Use of mRNA COVID-19 Vaccine After Reports of Myocarditis Among Vaccine Recipients: Update from the Advisory Committee on Immunization Practices — United States, June 2021. MMWR Morb Mortal Wkly Rep 2021;70(27):977-982.
20. Shimabukuro T, Advisory Committee on Immunization Practices (ACIP). COVID-19 Vaccine safety updates. 23rd June 2021
21. U.S. Department of Health and Human Services. Statement Following CDC ACIP Meeting from Nation's Leading Doctors, Nurses, Pharmacists and Public Health Leaders on Benefits of Vaccination. 2021.
22. Jain SS, Steele JM, Fonseca B, et al. COVID-19 Vaccination-Associated Myocarditis in Adolescents. Pediatrics 2021.
23. Medicines & Healthcare products Regulatory Agency. Coronavirus vaccine - weekly summary of Yellow Card reporting. 30th July 2021
24. Boehmer TK, Kompaniyets L, Lavery, AM, Hsu J, Ko JY, Yusuf H, Romano SD, Gundlapalli AV, Oster ME, Harris AE. Association Between COVID-19 and Myocarditis Using Hospital-Based Administrative Data — United States, March 2020–January 2021. Morbidity and Mortality Weekly Report (MMWR). September 3, 2021 / 70(35);1228–1232
25. Singer ME, Taub IB, Kaelber DC. Risk of Myocarditis from COVID-19 infection in people under 20: A population-based analysis. MedRxiv. 2021
26. Barda N, Dagan N, Ben-Shlomo Y, et al. Safety of the BNT162b2 mRNA Covid-19 Vaccine in a Nationwide Setting. *N Engl J Med* 2021; 385(12): 1078-90.
27. Nalbandian A, Sehgal K, Gupta A, et al. Post-acute COVID-19 syndrome. *Nat Med* 2021; 27(4): 601-15.
28. Ray STJ, Abdel-Mannan O, Sa M, et al. Neurological manifestations of SARS-CoV-2 infection in hospitalised children and adolescents in the UK: a prospective national cohort study. *Lancet Child Adolesc Health* 2021.
29. Douaud G LS, Alfaro-Almagro F, Arthofer C, Wang C, Lange F, Andersson JLR, Griffanti L. Brain imaging before and after COVID-19 in UK Biobank. *MedRxiv* 2021.
30. Public Health England. Weekly national Influenza and COVID-19 surveillance report: Week 36 report. 9 September 2021, 2021.
31. Public Health England. Hospitalisations by age, Englnd https://t.co/UC9VXodKXG?amp=1
32. Public Health England. COVID-19 Health Inequalities Monitoring for England (CHIME) tool. https://analytics.phe.gov.uk/apps/chime/
33. SAGE. Ninety-fifth SAGE meeting on COVID-19, 09 September 2021. https://assets.publishing.service.gov.uk/government/uploads/system/uploads/attachment_data/file/1017296/S1360_SAGE_95_minutes.pdf
34. Gov.uk. Coronavirus (COVID-19) in the UK. Cases in England. 2021. https://coronavirus.data.gov.uk/details/cases?areaType=nation&areaName=England

35. Clews ML. Covid 'surge' likely when schools return, expert warns. Tes. 2021
36 Wood A, Denholm R, Hollings S, et al. Linked electronic health records for research on a nationwide cohort of more than 54 million people in England: data resource. *BMJ* 2021; 373: n826.
37. Ministry of Health Israel. Explanation About the Effectiveness of the Vaccine for Coronavirus in Israel. 2021.
38. REACT-1 round 13 final report: exponential growth, high prevalence of SARS-CoV-2 and vaccine effectiveness associated with Delta variant in England during May to July 2021. 2021
39. Office for National Statistics. Coronavirus (COVID-19) latest insights: Vaccines 16 September 2021
40. Public Health Scotland. Daily Case Trends By Age and Sex. https://www.opendata.nhs.scot/dataset/covid-19-in-scotland/resource/9393bd66-5012-4f01-9bc5-e7a10accacf4
41. Swann OV, Holden KA, Turtle L, et al. Clinical characteristics of children and young people admitted to hospital with covid-19 in United Kingdom: prospective multicentre observational cohort study. *BMJ* 2020; 370: m3249.
42. Penner J, Abdel-Mannan O, Grant K, et al. 6-month multidisciplinary follow-up and outcomes of patients with paediatric inflammatory multisystem syndrome (PIMS-TS) at a UK tertiary paediatric hospital: a retrospective cohort study. *Lancet Child Adolesc Health* 2021; 5(7): 473-82.
43. Toraih EA, Hussein MH, Elshazli RM, et al. Multisystem inflammatory syndrome in pediatric COVID-19 patients: a meta-analysis. *World J Pediatr* 2021; 17(2): 141-51.
44. Gov.uk. ISARIC: Supplementary Information - comparison of children and young people admitted with SARS-CoV-2 across the UK in the first and second pandemic waves, 9 September 2021
45. Public Health England. Risk assessment for SARS-CoV-2 variant Delta: 23 July 2021, 2021.
46. Public Health England. SARS-CoV-2 variants of concern and variants under investigation in England. Technical briefings 15-17. 2021
47. Delahoy MJ, Ujamaa D, Whitaker M, O'Halloran A, Anglin O, Burne E et al. Hospitalizations Associated with COVID-19 Among Children and Adolescents — COVID-NET, 14 States, March 1, 2020–August 14, 2021. Morbidity and Mortality Weekly Report. 2021
48. Lopez Bernal J, Andrews N, Gower C, et al. Effectiveness of Covid-19 Vaccines against the B.1.617.2 (Delta) Variant. *N Engl J Med* 2021.
49. Rella SA, Kulikova YA, Dermitzakis ET, Kondrashov FA. Rates of SARS-CoV-2 transmission and vaccination impact the fate of vaccine-resistant strains. Scientific Reports. 11, 15729. 2021.
50. Gog JR, Hill EM, Danon L, Thompson RN. Vaccine escape in a heterogeneous population: insights for SARS-CoV-2 from a simple model. The Royal Society. 14 July 2021. 2021. https://doi.org/10.1098/rsos.210530
51. Hillis SD, Unwin HJT, Chen Y, et al. Global minimum estimates of children affected by COVID-19-associated orphanhood and deaths of caregivers: a modelling study. *Lancet* 2021; 398(10298): 391-402.
52. Office for National Statistics. Prevalence of ongoing symptoms following coronavirus (COVID-19) infection in the UK: 2 September 2021
53. Gurdasani D, Alwan NA, Greenhalgh T, et al. School reopening without robust COVID-19 mitigation risks accelerating the pandemic. *Lancet* 2021; 397(10280): 1177-8.
54. Centers for Disease Control and Prevention. Interim Public Health Recommendations for Fully Vaccinated People, 2021
55. World Health Organisation. COVID-19 Virtual Press conference transcript - 25 June 2021

Table 1: Data used to estimate hospitalisations, deaths, ICU admissions, vaccine associated myocarditis and long COVID

	Numbers	Percentage of cases
Total population 12-17 yr olds	3,918,373	
Boys	2,011,458	
Girls	1,906,915	
COVID-19 diagnosed cases 1/7/20 - 31/3/21	169,412	
Hospitalised	1,390	0.820%
ICU admissions	91	0.054%
Ventilated in ICU or outside ICU setting	75	0.044%
Died	11	0.006%
Long COVID (12 weeks) (2%,4% and 14% incidence)[6-10]	6,776 (4%)	2%, 4%, 14%
Vaccine-associated myocarditis/pericarditis		
Boys (12-17 yr old) (per million) [17-20]	6.72 (1st dose)<br>62.75 (2nd dose)	
Girls (12-17 yr old) (per million) [17-20]	0 (1st dose)<br>8.68 (2nd dose)	

These data have been extracted through linkage of electronic health records from multiple sources to assess the total number of children identified with a COVID-19 infection, and related hospitalisations, intensive care (ICU) admissions, ventilatory support and deaths (data accessed through the British Heart Foundation Data Science Centre).[15] Data sources include Second Generation Surveillance System (SGSS) national testing laboratory, primary care consultations in General Practice Extraction Service Data for Pandemic Planning and Research (GDPPR) using SNOMED-CT terms, Hospitalisations are identified from an admission in Hospital Episode Statistics (HES) Admitted Patient Care (APC) or Secondary Uses Service (SUS) containing COVID-19 ICD-10 diagnosis or an entry in COVID-19 Hospitalisations in England Surveillance System (CHESS). ICU admissions are identified by an entry in HES Critical Care (CC) or from CHESS. Ventilatory support is identified from CHESS, HES CC (basic or advanced respiratory support days > 0) and HES APC and SUS (OPCS-4 procedure codes for continuous positive airway pressure, non-invasive ventilation, invasive ventilation, intubation of trachea and extracorporeal membrane oxygenation). Deaths are identified from Office of National Statistics (ONS) Deaths Registry, with COVID-19 as a named cause of death or within 28 days of an individual's first COVID-19 event as well as from HES APC or SUS admissions with a discharge method or destination denoting death. Patients were included in the analyses if they resided in England, were alive on the study start date, registered with a primary care practice, had a valid pseudo-identifier for linkage and at least 28 days of follow up. We used the period from 1st July 2020-31st March 2021 to exclude the first wave of infections when few children were tested for COVID-19.[15]
Total number of 12-17yr olds taken from ONS population estimates for England: mid-2020 for persons by single year of age.

Figure 1: Time series of daily hospital admissions (7 day running average) with COVID-19 among 6-17 year olds from 1 April 2020 to 13 September 2021.

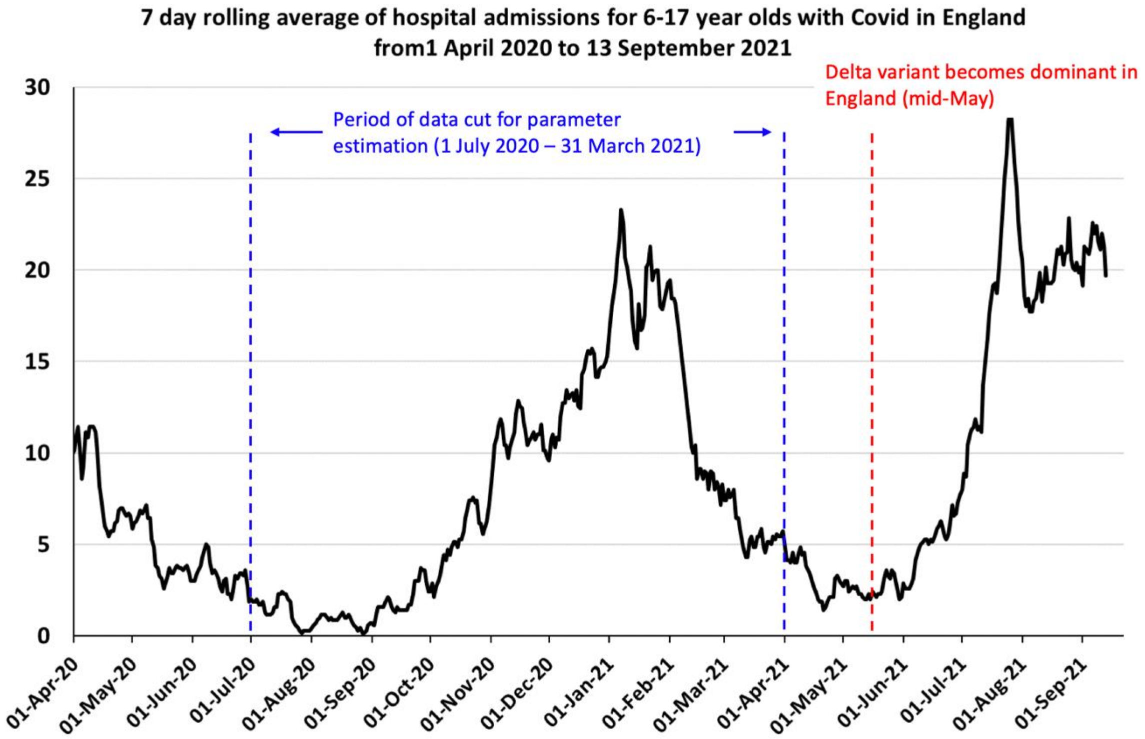

Data downloaded from https://api.coronavirus.data.gov.uk/v2/data?areaType=nation&areaCode=E92000001&metric=cumAdmissionsByAge&format=csv .

Figure 2: Risk-benefit of vaccination in adolescents at high and low incidence levels

A.

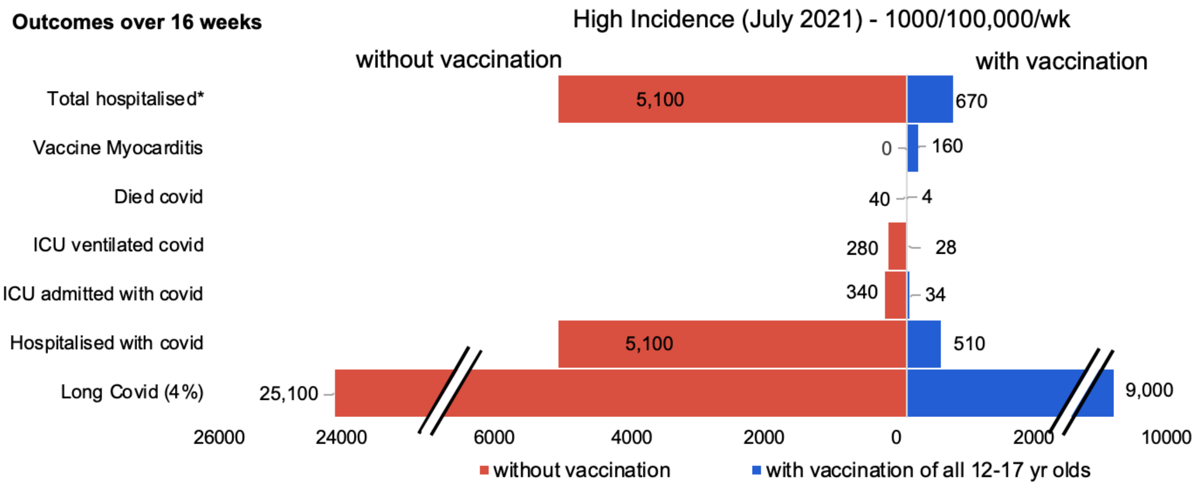

B.

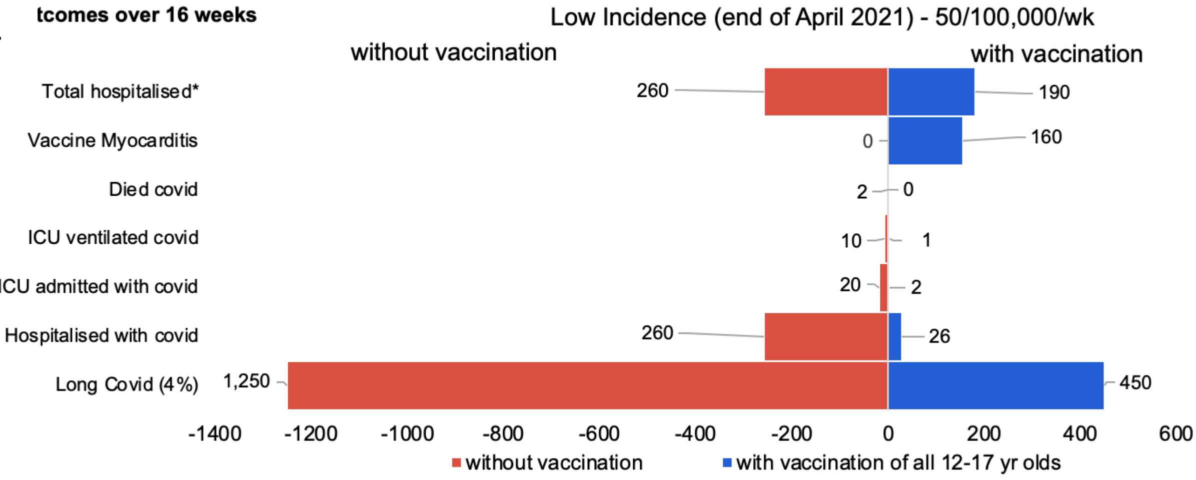

Fig 2A. and 2B. show a comparison of specific outcomes among adolescents aged 12-17 years of age calculated over a 16 week period assuming different levels of exposure with high incidence of 1000 per 100,000 per wk (reflecting the current case rates in this age group in England) and low incidence of 50 per 100,000 per week, corresponding to end of April 2021. **Note**: the scales for Fig 2A and 2B are different for ease of visualisation. In all cases, direct benefits of vaccination appear to considerably outweigh risks. Values above 50 have been rounded to the closest ten. Myocarditis here refers to both vaccine related myocarditis and pericarditis. We show long COVID estimates assuming an incidence rate of 4% - see results section for equivalent estimates of 2% and 14% incidence.

*total hospitalised considers hospitalisations from COVID-19 and vaccine related myocarditis/pericarditis (assuming a worst-case scenario that all cases of myocarditis are hospitalised)

Figure 3: Hospitalisations* and deaths averted by vaccination in adolescents at different incidence levels

A.

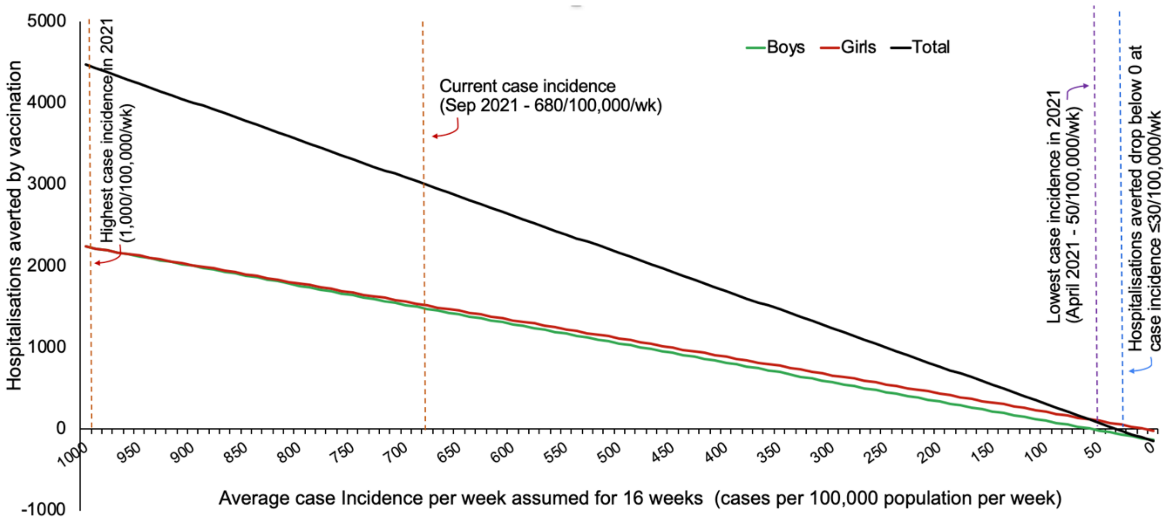

B.

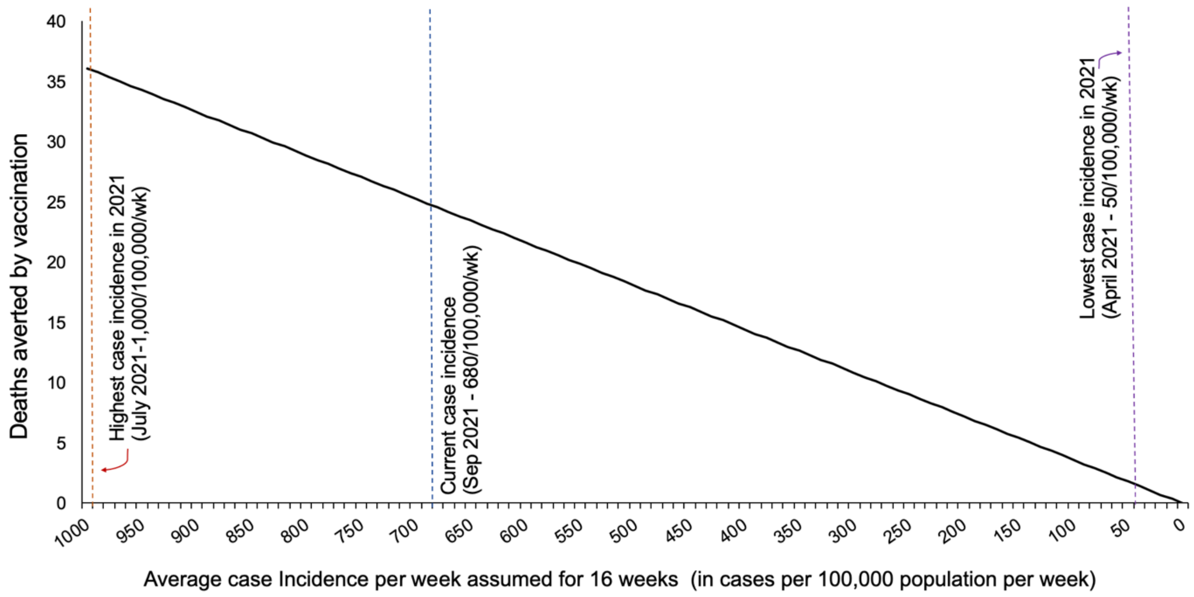

Fig 3A. and 2B. depict the number of hospitalisations*, and deaths averted as a function of case incidence among 12-17 year olds over a 16 week period. For hospitalisations, we represent these separately for boys and girls to account for the differing rate of vaccine-related myocarditis. Myocarditis here refers to both vaccine related myocarditis and pericarditis.

*total hospitalised considers hospitalisations from COVID-19 and vaccine related myocarditis/pericarditis (assuming a worst-case scenario that all cases of myocarditis are hospitalised)
